# Isolation and identification of major bacteria from three Ethiopian rift valley lakes live and processed fish, and water samples: implications in sanitary system of fish products

**DOI:** 10.1186/s12917-022-03508-w

**Published:** 2022-12-14

**Authors:** Guta Dissasa, Brook Lemma, Hassen Mamo

**Affiliations:** 1 Institute of Biotechnology, Addis Ababa University, Addis Ababa, Ethiopia; 2 Department of Zoological Sciences, College of Natural and Computational Sciences, Addis Ababa University, Addis Ababa, Ethiopia; 3 Department of Microbial, Cellular and Molecular Biology, College of Natural and Computational Sciences, Addis Ababa University, Addis Ababa, Ethiopia

**Keywords:** Fish bacteria, Gram-negative bacteria, Physicochemical parameters, Rift-valley lakes

## Abstract

Bacterial pathogens are a great threat to fish production. Gram-negative bacteria are among the major bacterial fish pathogens and are zoonotic with the potential to infect humans. This cross-sectional study was conducted to isolate and identify major gram-negative bacteria from live and processed fish, and water samples from Lakes Hawassa, Langanoo and Ziway. A total of 674 different types of samples: 630 tissue samples (210 samples for each intestine, Kkidney and liver collected from 210 live fish (*Oreochromis niloticus*, *Cyprinus carpio* and *Clarias gariepinus*), 20 processed fish samples from lake Ziway fish processing center and 24 lake water samples were included in the study from each lake. The mean values of pH, temperature, dissolved oxygen and nitrate in all water samples were within the normal range at which most freshwater fish species become non-stressed. Of a total of 674 samples included in the study, bacteria were isolated from 154(22.8%) samples with significant difference (*P* < 0.05) observed in some isolates with respect to sample origin. Of these 154 isolates, 103(66.8%) isolates were gram-negative bacteria consisting of 15 species based on morphology and a range of biochemical tests. From live fish samples, *Escherichia coli *was the dominant species with 15 isolates followed by *Edwardsiella tarda *(12), *Salmonella Paratyphi *(10), *Salmonella Typhi *(9),  *Shigella dysenteriae* (7), *Shigella flexneri *(7), *Klebsiella pneumonia *(7), *Enterobacter aerogenes *(6), *Enterobacter cloacae *(5), *Pseudomonas aeruginosa *(5), *Vibrio parahemolyticus *(5), *Aeromonas sobria* (4), *Citrobacter freundii *(4), *Citrobacter koseri* (4) and *Plesiomonas shigelloides* (3). The detection of the common fecal coliforms (*E. coli*, *K. pneumoniae * and *E. aerogenes*) and *Salmonella spp*. in processed fish indicates the potential danger of passage of pathogenic bacteria and/or their poisons to humans via infected and/or contaminated fish products. Human infection by pathogenic fish bacteria and food poisoning is possible through contamination of fish product in fish production chain due to inadequate handling, poor hygiene and contact with contaminated water. Therefore, producers, consumers and all other stakeholders need to be cautious during handling, processing and consumption of fish harvested from the study lakes.

**Supplementary Information:**

The online version contains supplementary material available at 10.1186/s12917-022-03508-w.

## Background

Fish plays an important role in the human diet with an ever-growing need globally. World fish production has increased dramatically during the past 60 years, to around 179 million tons in 2018 with a value of $401 billion and global fish consumption also increased from 9.0 kg per capita in 1961 to 20.5 kg in 2018 [1]. This is a tremendous change in the fishery industry. Ethiopia depends on its inland lakes and rivers for fish production, and has over 200 edible fish species with annual fish production potential of about 94,500 thousand tons [2] although the potential remains much higher.

From a broader perspective, fish is among the top components of aquatic biodiversity and is closely connected with several sectors of human socioeconomic life. However, fish health is a factor of aquatic ecosystem dynamics. Pathogenic fish bacteria, among other variables, disturb ecosystem dynamics and affect aquatic biodiversity and fish wellbeing; erode the fishery industry and food security. Moreover, fish consumption is associated with some serious bacterial infections mainly due to poor sanitary facilities and practices around water systems, unhygienic conditions in fishing and fish production chain. In particular, gram-negative bacteria like *Aeromonas* spp., *Flavobacterium* spp., *Pseudomonas* spp., *Edwardsiella* spp., *Vibrio* spp., *Acinetobacter* spp. and *Plesiomonas shigelloides* are a great threat to fish production [3, 4].

Most of these bacterial fish pathogens are zoonotic with a potential to infect humans, and some of them are serious [5]. Besides, fish pathogenic bacteria influence fish population with substantial concerns for aquatic biodiversity and fish industry, and food security. Studies on fish microbial community provide a clue about the hygienic status of the environment as water quality and fish diseases are closely linked [6]. Detection of fish pathogenic bacteria including novel ones, or change in the water microflora is an important indicator of environmental contamination. This is due to negative ecological changes in the natural fish habitat. Progressive degradation of aquatic ecosystems is due to human activities associated with urbanization, industrialization, and agriculture resulting in the release of sewages of different nature and origin [7]. Additional factor that contributes to the emergence of novel fish pathogenic bacteria is unmanaged use of antibiotics and disinfectants [8]. Non-human factors like climate change and unpredictable natural disasters seriously affect water bodies and all life therein [9, 10].

The risk of contamination of water bodies, particularly inland closed systems like the Ethiopian rift-valley lakes, is particularly increasing due to clearly visible extensive nearby development activities, urbanization and to certain extent frequent visits for various reasons including international ecotourism. Therefore, assessment of the physicochemical components of aquatic ecosystems and their bacterial community including inside resident fish is essential to monitor fish health, product quality and related potential environmental and public health challenges. Reports on bacterial pathogens in freshwater fish, water physicochemical parameters and bacteriological quality of the Ethiopian rift-valley lakes are scant. Hence, this study was aimed to assess the occurrence, distribution, prevalence and identity of major gram-negative enteric bacteria from live-caught and commercial processed fish product of three common fish species—Nile tilapia (*Oreochromis niloticus*), common carp (*Cyprinus carpio*) and catfish (*Clarias gariepinus*)—and water samples from three Ethiopian rift-valley (inland) Lakes Lake Hawassa, Lake Langano and Lake Ziway in Ethiopia.

## Methods

### The study area

**Fig. 1:**
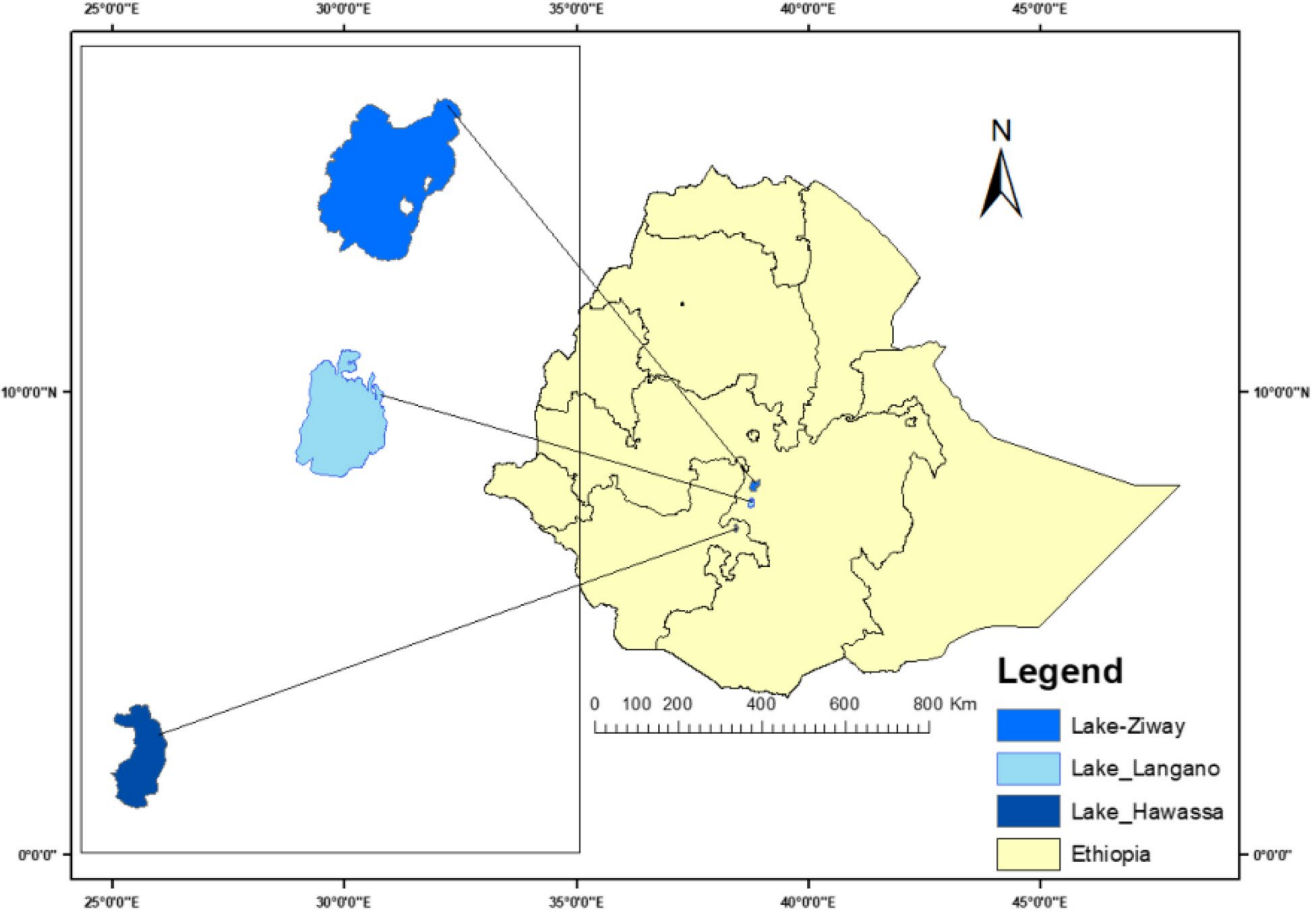
The study area

The study was on three Ethiopian rift-valley Lakes, Lake Ziway, Lake Langano and Lake Hawassa, which are well-known for their common fish catches and tourist destinations. These Lakes are some 163, 200 and 275 km to the south of Addis Ababa respectively (Fig. 1). Morphometric and other characteristics of the Lakes are shown in Supplementary Table 1 with information sources from https://latitudelongitude.org/et [11], World Lake Database [12], Wood and Talling 1988 [13].

### Sample size

The sample size used to estimate the prevalence of bacterial infections in live fish samples was determined using the formula *n* = ${Z}^{2}P(1-P))/{d}^{2}$ by Daniel and Cross (2013) [14], where 'n' is the sample size, 'Z' the confidence interval (1.96), 'P' the average prevalence estimate of 16.5% calculated from a review report in Ethiopia [15], and 'd' the expected error (0.05).

### Study design and sampling strategy

The study design was cross-sectional and its period was from February to August 2021. For sampling, the three Lakes were selected purposively that the Lakes are known for their common fish catches and from which fish are continuously harvested for subsistence and cash [16]. Different sampling points of each Lake were used to measure physicochemical parameters, and collect live fish and water samples.

Overall, 12 sampling sites, four from each Lake, were selected guided by the available information. The sampling points are considered major fishing grounds along the shorelines. Accordingly, *Amora Gedel* (S1), *Haile resort area* (S2), *Inlet of Tikurwuha River* (S3) and *Referral Hospital area* (S4) were sites of Lake Hawassa. *Dole* (S1), *O’etu* (S2), *Wabishebelle* (S3) and *Yakona* (S4) were sites of Lake Langano﻿; and *Abosa* (S1), *Bochessa* (S2), *Cafeteria* (S3) and *Wasiko* (S4) were sites selected at Lake Ziway. These sites were located at different directions of the Lakes and were about 3-7 km apart. Thus, the sites were assumed to represent each lake.

### Water samples for nutrient and bacteriological analysis

The on-spot-checkable water chemistry parameters were tested in situ using routine methods [17]*.* Temperature, electrical conductivity (EC) and dissolved oxygen (DO) were measured using temperature meter (YSI model 33 S-C-T meter, USA), conductivity meter (JENWAY, Multi-3410, UK) and multi parameter (Multi-3410, Germany) respectively. The pH was recorded using a digital pH meter (HI-99130, Italy).

Surface water samples from each sampling point were collected into sterile glass bottles (100 ml) as per standard sampling procedure for lake or stream surface water chemistry for nutrient content testing [18] and bacteriological analysis [19, 20]. The water samples were kept dark, packed, and refrigerated. For live fish sampling, a random sampling technique was applied to represent the three target fish types (the tilapia, common carp and catfish). In case of external disease occurrence, diseased fish were purposively selected. The fish were drawn from the peripheral, mid and central regions of the sampling points using fishing boats and gears of local fishers.

### Fish samples

The live fish samples were inspected for external abnormalities, and were grouped into apparently healthy and clinically sick. The fish were euthanized by cervical dislocation in the fish's normal water without using anesthesia. Trained individuals practiced the technique using appropriate equipment. Cervical dislocation is among the methods recommended for fish sacrifice as it is relatively simple and effective for not big fish [21]. As our fish were small to medium in size, they were killed by inserting a rod or thumb into the mouth, holding with the opposite hand and displacing it dorsally. Death was recognized by cessation of movement, and confirmed by cessation of respiration (opercular movement) and cessation of heartbeat (palpation); and finally by destruction of the brain. The samples were separately packed in sterile plastic bags and were shipped to *Batu Fisheries and Other Aquatic Life Research Center* located at Batu town (formerly Ziway), near Lake Ziway. The fish were dissected under aseptic conditions using a sterile dissecting scissor by following established protocol [22, 23] and standard operating procedures of bacteriology [19, 20].

Examination of the internal fish anatomy was made by observing for any abnormalities including position, size, color and other signs of damage. The digestive tract, gonads and visceral organs were removed by cutting the esophagus and disconnecting them from the kidneys. Using sterile scalpel blade or forceps, tissue samples of the kidney, intestine and liver were aseptically transferred to sterile bottles (100 ml) with physiological saline, and were homogenized for bacteriological analysis [19].

Similarly, about 10 g muscle was cut from processed fish in Batu town fish processing center using a sterile knife, kept in sterile universal bottles (100 ml), and homogenized with 10 ml physiological saline solution. From the homogenized samples of both live and processed fish, 1 ml aliquot was drawn and further homogenized in a clean, dry sterile beaker containing 9 ml of distilled water to have 1:10 dilution [20].

Finally, all the three sample types (water, live fish and processed fish) were packaged and transported on ice to Health Biotechnology Laboratory of Institute of Biotechnology, Addis Ababa University, and were stored at 4 °C for nutrient and bacteriological analysis.

### Water nutrient content analysis

The nitrate content of the transported water samples was determined using the sodium salicylate method and phosphate by ascorbic acid method using UV–visible spectrophotometer (Lambda, CE1021, and Australia).

### Bacteria culture and colony morphology

All bacteriological experiments were performed following Society of American Bacteriologists *Manual of microbiological methods* [19], *Bergey’s manual of determinative bacteriology* [20] and the respective media manufacturer's instructions. It was under complete aseptic conditions. All incubations were for 24 h at 37 °C.

First, sample swabs were spread or streaked across nutrient agar medium (Oxoid, England) and incubated under aerobic condition. Then, specific colonies were picked-up and inoculated on selective and differential media Xylose Lysine Deoxycholate (XLD) agar (HIMEDIA, India), and incubated further. Suspected bacterial colonies were picked-up, inoculated into tryptone soy broth (HIMEDIA, India), and incubated. Colony morphology like form, elevation, margin, surface and pigmentation were examined. Colony color was determined by visual inspection of bacterial cell suspensions using fresh culture. The colonies were screened microscopically using simple and differential (gram) staining. The morphologically presumptively identified isolates were stored at -20 °C in 50% glycerol (Fine Chemical, Ethiopia) using 1.8 ml cryovials (IMEC, China) for biochemical identification.

### Biochemical characterization

All the media used in the tests were from HIMEDIA, India. The tests began with inoculating respective media with 24-h-old pure culture colonies. All incubations were at 37 °C for 24 h and the expected color changes confirmed test positivity. Briefly, indole production was tested by inoculating 10 ml of Dev tryptophan broth, incubating and adding 2–3 drops of indole reagent. Methyl red (MR) test was conducted by inoculating 10 ml of MR Voges-Proskauer (MR-VP) medium, incubating, and adding 2–3 drops of 0.05% MR. Voges-Proskauer (VP) test was done by inoculating 10 ml of MR-VP medium, incubating, and adding 2–3 drops of 5% a-nephtol followed by 40% of KOH and shaking and leaving it open for an hour.

For catalase test, a small amount of bacterial colony was transferred to clean glass slide using a sterile loop and a drop of hydrogen peroxide was added, and the formation of bubbles was checked for. For citrate utilization test, Simmons citrate agar slant was inoculated and incubated. Hydrogen sulfide or triple sugar iron (TSI) test was done by inoculating TSI by first stabbing through the center of the medium to the bottom of the tube and then streaking the surface of the agar slant, and incubating. Similarly, urea production was tested using Christensen’s Urea Agar slant. Sugar fermentation test was conducted using sugar broth medium prepared by mixing 1 g peptone, 0.3 g meat extract, 0.5 g table salt, 0.5 g sugar and 0.008 g phenol indictor in 100 ml distilled water. Three tubes having three different sugars (glucose, sucrose, lactose) in the broth medium were inoculated, and incubated.

### Data analysis

Bacterial infection status of the different sample types was determined and the proportion of infected samples/isolates was compared between various categories using the Chi-squared test. Bacterial species diversity in different sample types was compared using one-way analysis of variance (ANOVA). Statistical analysis was performed using IBM SPSS software version 20 (IBM, Chicago, USA) and *p* < 0.05 was considered statistically significant.

## Results

### Sample type and total number

The sampled live-caught fish were 210. Of these, 42(20%) had evident external health problems and the rest were apparently healthy both externally and after dissection. In terms of species, 105 were tilapia (*O. niloticus*)*,* 60 common carp (*C. carpio*) and 45 catfish (*C. sgariepinus*). Overall, 90, 50 and 70 of the fish were from Lake Ziway, Langanoo and Hawassa respectively. The diseased fish were 23 tilapia (8 from Lake Hawassa, 2 from Langanoo and 13 from Ziway) plus 19 carps (12 from Lake Ziway and 7 from Hawassa). Intestine, kidney and liver tissue samples were taken from each fish making the number of samples of live fish origin 630. The size of processed fish samples was 20. The total number of water samples was 36. That is, duplicate samples from each 4 sampling points of each lake making 24 samples for bacteriological analysis, and additional 12 samples from each point of each lake for water chemistry. Hence, the overall sample number undergone bacteriological analysis was 674.

### Physicochemical parameters

The mean pH values of the three Lakes were nearly similar ranging 7.91–8.68. The temperature of Lake Hawassa was higher (25.8 °C) than that of the other two that had the same measure. Lake Ziway had the lowest OD (3.93 ± 0.18 mg/ml) and Hawassa the highest (6.27 ± 0.90 mg/ml). The highest EC (µS/cm) was that of Lake Hawassa (1673.5 ± 0.48) followed by Langanoo (1447 ± 5.70) and the least Ziway (289.5 ± 14). It is notable that the EC of Lake Ziway was fivefold below that of Hawassa. All records including for nitrate were highest in Lake Hawassa and least in Ziway except for pH. The highest mean phosphate level was for Lake Langano followed by Hawassa and Ziway with all the three lakes having higher values than the standard limit. However, all the measured physicochemical parameters of the three Lakes (Supplementary Table 2) were at optimum level or at least within the tolerance range of most freshwater fish species.

### Bacterial isolates

From the 674 samples, 154(22.8%) were positive for bacteria (51 g-positive and 103 g-negative isolates). The gram-negative bacteria were further tested and identified into 15 species. All isolates were short rods and non-spore formers (Supplementary Fig. 1). Colonies of the isolates had different form, elevation, margin, surface, colour and optical characteristics (Supplementary Fig. 2).

The prevalence of gram-negative bacteria among water, processed fish and live-caught fish samples was 50% (12/24), 45% (9/20) and 39% (82/210) respectively. The difference was statistically significant (*p* < 0.0001). *Escherichia coli* was the dominant species with 15 isolates (12 from live-caught fish, 2 processed fish, 1 water sample) followed by 12 *Edwardsiella tarda* (10 live fish, 0 processed fish, 2 water sample)), 10 *Salmonella Paratyphi* (7 live fish, 2 processed fish, 1 water sample) and 9 *Salmonella typhi* (7 live fish, 2 processed fish, 0 water sample). The other gram-negative bacteria were *Shigella dysenteriae*, *Shigella flexneri, Klebsiella pneumonia, Enterobacter cloacae*, *Pseudomonas aeruginosa, Vibrio parahemolyticus*, *Aeromonas sobria*, *Citrobacter freundii*, *Citrobacter koseri, Enterobacter aerogenes* and *Plesiomonas shigelloides*.

While all of the above species have been isolated from live fish samples, *E. aerogenes, E. cloacae, P. shigelloides* and *S. typhi* were not detected in any of the water samples. Only *E. coli, K. pneumoniae* and *E. aerogenes* (the common fecal coliforms) and *Salmonella* spp. were detected in processed fish samples. *E. coli*, *K. pneumonia* and *S. Paratyphi* were detected in all the three sample types. Bacteria isolated from water samples, but not from processed fish, were *A. sobria*, *C. freundii*, *C. koseri, E. tarda, P. aeruginosa, S. dysenteriae, S. flexneri* and *V. parahemolyticus*. Conversely, *E. aerogenes* and *S. typhi* were identified from processed fish but not from water samples (Table 1). The distribution of individual bacterial species was not statistically significant with respect to sample type.

**Table 1:** The number and proportion of samples positive for Gram-negative bacteria species among water (*N* = 24) and live fish (*N* = 210) samples collected from Lakes Hawassa, Langanoo and Ziway, and processed fish samples (*N* = 210) from Batu town

Bacteria spp	Sample type (*n* = 154)			
	Water, n(%)	Live fish, n(%)	Processed fish, n(%)	Total
*A. sobria*	1(5.9)	3(2.4)	0(0.0)	4(2.6)
*C. freundii*	1(5.9)	3(2.4)	0(0.0)	4(2.6)
*C. koseri*	1(5.9)	3(2.4)	0(0.0)	4(2.6)
*E. tarda*	2(11.8)	10(8.1)	0(0.0)	12(7.8)
*E. aerogenes*	0(0.0)	4(3.2)	1(7.7)	5(3.2)
*E. cloacae*	0(0.0)	5(4.0)	0(0.0)	5(3.2)
*E. coli*	1(5.9)	12(9.7)	2(15.4)	15(9.7)
*K. pneumoniae*	1(5.9)	4(3.2)	2(15.4)	7(4.5)
*P. shigelloides*	0(0.0)	3(2.4)	0(0.0)	3(1.9)
*P. aeruginosa*	1(5.9)	4(3.2)	0(0.0)	5(3.2)
*S. paratyphi*	1(5.9)	7(5.6)	2(15.4)	10(6.5)
*S. typhi*	0(0.0)	7(5.6)	2(15.4)	9(5.8)
*S. dysenteriae*	1(5.9)	6(4.8)	0(0.0)	7(4.5)
*S. flexneri*	1(5.9)	6(4.8)	0(0.0)	7(4.5)
*V. parahemolyticus*	1(5.9)	4(3.2)	0(0.0)	5(3.2)
Total	12(50)	82(39.1)	9(45)	< 0.0001

### Bacteria distribution by live fish tissue

The highest number of isolates (37(17.6%)) were recovered from the intestine followed by the liver (26(12.4%)) and kidney 19(9.1%) with statistically significant difference (*p* < 0.0001). The most frequently isolated bacterium from live fish was *E. coli* with 5 isolates (2.4%) in the intestine and 5(3.6%) in the liver (Table 2). *E. aerogenes* was the most frequent isolate from the kidney (2.1%). The least frequently isolated bacterium from live fish was *P. shigelloides* (1.4%). There was no significant difference in the distribution of individual bacterial species in the three fish tissues except for *V. parahemolyticus* whose prevalence in the intestine was significantly higher (*p* = 0.048).

**Table 2:** Distribution of bacteria in the kidney, liver and intestine of live fish caught from Lakes Hawassa, Langanoo and Ziway (*N* = 210)

Isolated bacteria	Intestine, n(%)	Kidney, n(%)	Liver, n(%)	Total, n(%)	*P*-value
*A. sobria*	1(0.5)	-	2(1.4)	3(1.4)	0.064
*C. freundii*	2(0.9)	-	1(0.5)	3(1.4)	0.244
*C. koseri*	2(0.9)	1(0.5)	-	3(1.4)	0.064
*E. tarda*	4(1.9)	2(0.9)	4(1.9)	10(4.8)	0.332
*E. aerogenes*	1(0.5)	3(2.1)	1(0.5)	5(2.4)	0.394
*E. cloacae+*	2(0.9)	2(0.9)	1(0.5)	5(2.4)	0.630
E. coli*	5(2.4)	2(0.9)	5(3.6)	12(5.7)	0.104
*K. pneumoniae*	1(0.5)	2(0.9)	1(0.5)	4(1.9)	0.579
*P. shigelloides*	1(0.5)	2(0.9)	-	3(1.4)	0.244
*P. aeruginosa*	2(0.9)	1(0.5)	1(0.5)	4(1.9)	0.702
*S. paraTyphi*	3(1.4)	2(0.9)	2(0.9)	7(3.3)	0.770
*S. Typhi*	4(1.9)	1(0.5)	2(0.9)	7(3.3)	0.406
*S. dysenteriae*	3(1.4)	-	3(1.4)	6(2.9)	0.262
*S. flexneri*	3(1.4)	-	3(1.4)	6(2.9)	0.166
*V. parahemolyticus*	3(1.4)	1(0.5)	-	4(1.9)	0.048^*^
Total	37(17.6)	19(9.1)	26(12.4)	82(39.1)	< 0.0001^*^

*As* *Aeromonas sobria, Cf* *Citrobacter freundii, Ck* *Citrobacter koseri, Ct* *Edwardsiella tarda, Ea* *Enterobacter aerogenes, Ec*^+^ Enterobacter cloacae, Ec*i *Escherichia coli, Kp* *Klebsiella pneumonia, Ps* *Plesiomonas shigelloides, Pa* *Pseudomonas aeruginosa, Sp* *Salmonella Paratyphi, St* *Salmonella typhi, Sd* *Shigella dysenteriae, Sf* *Shigella flexneri, Vp* *Vibrio parahemolyticus*

^*^ means significant at 5% level

### Bacteria distribution by fish species

Except *A. sobria*, all other isolates were recovered from tilapia (*O. niloticus*) live fish catches. Similarly, all other bacterial species were isolated from catfish (*C. gariepinus*) with the exception of *P. shigelloides.* However, *C. freundii, C, koseri* and *V. parahemolyticus* were not isolated from carps. Significant variation (*p* < 0.0001) was recorded regarding the prevalence of bacterial infection in the three fish species (Table 3).

**Table 3:** Number (percentage) of bacterial species isolated from three fish species caught from Lakes Hawassa, Langanoo and Ziway

Isolated bacteria	Carps *(Cc) N* = 60	Tilapia (*On) N* = 105	Catfish (*Cg) N* = 45	Total *n* = 210	*P*-value
*A. sobria*	2(3.3)	-	1(2.2)	3(1.4)	0.244
*C. freundii*	-	2(1.9)	1(2.2)	3(1.4)	0.064
*C. koseri*	-	1(1.4)	2(4.4)	3(1.4)	0.244
*E. tarda*	3(5)	4(3.8)	3(6.7)	10(4.8)	0.770
*E. aerogenes*	1(1.7)	1(1.4)	3(6.7)	5(2.4)	0.216
*E. cloacae+*	1(1.7)	1(1.4)	3(6.7)	5(2.4)	0.079
E. coli*	3(5)	6(5.7)	3(6.7)	12(5.7)	0.084
*K. pneumoniae*	1(1.7)	2(1.9)	1(2.2)	4(1.9)	0.579
*P. shigelloides*	2(3.3)	1(1.4)	-	3(1.4)	0.244
*P. aeruginosa*	1(1.7)	2(1.9)	1(2.2)	4(1.9)	0.702
*S. paratyphi*	2(3.3)	3(2.9)	2(4.4)	7(3.3)	0.422
*S. typhi*	2(3.3)	3(2.9)	2(4.4)	7(3.3)	0.579
*S. dysenteriae*	2(3.3)	3(2.9)	1(2.2)	6(2.9)	0.422
S. *flexneri*	3(5)	1(1.4)	2(4.4)	6(2.9)	0.125
*V. parahemolyticus*	-	2(1.9)	2(4.4)	4(1.9)	0.125
Total	23(38.3)	32(30.5)	27(60.0)	82(39.1)	< 0.0001**

*On* *O. niloticus*, *Cg* *C. gariepinus*, Cc C. carpio^**^ means significant at 5% level, *As* *Aeromonas sobria*, *Cf* *Citrobacter freundii*, *Ck* *Citrobacter koseri*, *Ct* *Edwardsiella tarda*, *Ea* *Enterobacter aerogenes*, *Ec*^+^ *Enterobacter cloacae*, Ec* *Escherichia coli, Kp* *Klebsiella pneumonia*, *Ps* *Plesiomonas shigelloides*, *Pa* *Pseudomonas aeruginosa*, *Sp* *Salmonella ParaTyphi, St* *Salmonella Typhi*, *Sd* *Shigella dysenteriae, Sf* *Shigella flexneri, Vp* *Vibrio parahemolyticus*

### Bacteria distribution in live fish from the three lakes

The highest number of isolates were from Lake Hawassa (34(48.6%)) followed by Ziway (35(43.8%)) and Langanoo the least with only 13(21.7%). The prevalence of bacterial infection in the three Lakes was statistically significant (*p* < 0.0001). All the fifteen bacterial species (100%) isolated in this study were in Lake Hawassa fish samples. Similarly, all of the species were also isolated from Lake Ziway fish samples except *C. freundii*. On the other hand, only 9(60.0%) of the 15 species were detected from Lake Langano fish samples (Table 4). The data shows the highest bacterial species composition in Lake Hawassa and the least in Langanoo.

**Table 4:** Number (percentage) of bacteria isolated from live-caught fish samples from Lakes Hawassa, Langanoo and Ziway

Isolated bacteria	Lake Hawassa *N* = 70	Lake Langano *N* = 60	Lake Ziway *N* = 80	Total *N* = 210	*P*-value
*A. sobria*	2(2.9)	-	1(1.3)	3(1.4)	0.064
*C. freundii*	2(2.9)	1(1.7)	-	3(1.4)	0.187
*C. koseri*	2(2.9)	-	1(1.3)	3(1.4)	0.187
*E. tarda*	5(7.1)	2(3.3)	3(3.8)	10(4.8)	0.296
*E. aerogenes*	1(1.4)	1(1.7)	3(3.8)	5(2.4)	0.394
*E. cloacae+*	2(2.9)	-	3(3.8)	5(2.4)	0.098
E. coli*	5(7.1)	2(3.3)	5(6.3)	12(5.7)	0.146
*K. pneumoniae*	2(2.9)	-	2(2.5)	4(1.9)	0.125
*P. shigelloides*	1(1.4)	1(1.7)	2(2.5)	4(1.9)	0.579
*P. aeruginosa*	1(1.4)	-	2(2.5)	3(1.4)	0.302
*S. paratyphi*	3(4.3)	1(1.7)	3(3.8)	7(3.3)	0.296
*S. typhi*	2(2.9)	3(5)	2(2.5)	7(3.3)	0.729
*S. dysenteriae*	2(2.9)	1(1.7)	3(3.8)	6(2.9)	0.579
*S*. *flexneri*	3(4.3)	1(1.7)	2(2.5)	6(2.9)	0.536
*V. parahemolyticus*	1(1.4)	-	3(3.8)	4(1.9)	0.072
Total	34(48.6)	13(21.7)	35(43.8)	82(39.1)	< 0.0001^**^

*As* *Aeromonas sobria*, *Cf* *Citrobacter freundii*, *Ck* *Citrobacter koseri*, *Ct* *Edwardsiella tarda*, *Ea* *Enterobacter aerogenes*, *Ec*^+^ *Enterobacter cloacae*, Ec* *Escherichia coli*, *Kp* Klebsiella pneumonia, *Ps* Plesiomonas shigelloides, *Pa* Pseudomonas aeruginosa, *Sp* Salmonella ParaTyphi, *St* Salmonella Typhi, *Sd* Shigella dysenteriae, *Sf* Shigella flexneri, *Vp* Vibrio parahemolyticus

^*^^*^ means significant at 5% level

### Bacteria distribution among clinically sick fish

Out of 42 clinically diseased tilapia and catfish samples from the three lakes, 36(85.7%) were positive for bacterial infection. These were 19 tilapias (11 from Lake Hawassa and 8 from Lake Ziway), and 17 carps (8 from Lake Hawassa and 9 from lake Ziway). No bacteria were isolated from diseased fish caught from Lake Langano (Supplementary Table 3). *E. tarda* was isolated from 5 tilapias and 2 carps in Lake Hawassa with overall prevalence of 19.4% which was significantly higher than the occurrence of any other species among sick fish from both Lakes (*p* = 0.046).

### Bacteria distribution in live fish by sampling site

Majority of Lake Hawassa isolates were from *S1* (22.9%) and *S4* (18.6%). In Lake Langano, most isolates were from *S2* (10%). Similarly, most of the isolates were from sampling points *S3* (18.8%) and *S1* (13.8%) of Lake Ziway. The distribution of *A. sobria*, *C. freundii*, *C. koseri, E. cloacae, E. coli, P. aeruginosa* and *V. parahemolyticus* varied significantly across live fish sampling sites of each Lake (Table 5).

**Table 5:** Number (percentage) of bacteria isolated from fish sampled from four different sites (S1-S4) of Lakes Hawassa, Langanoo and Ziway

Isolated bacteria	Lake Hawassa *n* = 70				Lake Langano *n* = 60				Lake Ziway *n* = 80				Total *n* = 210	*P*-value
	S1	S2	S3	S4	S1	S2	S3	S4	S1	S2	S3	S4		
*A. sobria*	1(1.4)	-	-	1(1.4)	-	-	-	-	-	-	1(1.3)	-	3(1.4)	0.001^*^
*C. freundii*	2(2.9)	-	-	-	-	-	1(1.7)	-	-	-	-	-	3(1.4)	0.001^*^
*C. koseri*	2(2.9)	-	-	-	-	-	-	-	1(1.3)	-	-	-	3(1.4)	0.001^*^
*E. tarda*	3(4.3)	-	-	2(2.9)	-	2(3.3)	-		-	-	2(2.5)	1(1.3)	10(4.8)	0.096
*E. aerogenes*	-	-	-	1(1.4)	-	-	1(1.6)	-	1(1.3)	1(1.3)	-	1(1.3)	5(2.4)	0.206
*E. cloacae*	1(1.4)	-	1(1.4)	-	-	-	-	-	1(1.3)	1(1.3)	1(1.2)	-	5(2.4)	0.003^*^
*E. coli*	2(2.9)	1(1.4)	-	2(2.9)	1(1.7)	-	-	1(1.7)	2(2.5)	-	1(1.3)	2(2.5)	12(5.7)	0.016^*^
*K. pneumoniae*	-	1(1.4)	-	1(1.4)	-	-	-	-	1(1.3)	-	1(1.3)		4(1.9)	0.153
*P. shigelloides*	-	-	-	1(1.4)	-	1(1.7)	-	-	2(2.5)	-		-	3(1.4)	0.287
*P. aeroginosa*	1(1.4)	-	-	-	-	-	-	-	-	-	2(2.5)	-	4(1.9)	0.003^*^
*S. paratyphi*	2(2.9)	1(1.4)	-	-	-	-	1(1.7)	-	2(2.5)	-	1(1.3)	-	7(3.3)	0.364
*S. typhi*	-	1(1.4)	-	1(1.4)	1(1.7)	1(1.7)		1(1.7)	1(1.3)	1(1.3)	-	-	7(3.3)	0.068
*S. dysenteriae*	1(1.4)	-	-	1(1.4)	-	1(1.7)	-	-	1(1.3)	-	2(2.5)	-	6(2.9)	0.413
S. *flexneri*	-	1(1.4)	-	2(2.9)	-	1(1.7)	-	-	-	-	2(2.5)	-	6(2.9)	0.158
*V. parahemo*	-	-	-	1(1.4)	-	-	-	-	-	-	2(2.5)	1(1.3)	4(1.9)	0.007^*^
Total	15(21.4)	5(7.1)	1(1.4)	13(18.6)	2(3.3)	6(10)	3(5)	2(3.3)	12(15)	3(3.8)	15(18.8)	5(6.3)	82(39.1)	

*S1* sample site 1, *S2* sample site 2, *S3* sample site 3, *S4* sample site 4, *n* number of isolates

^*^ statistically significant;—not isolated, *V. parahem*, *V. parahemolyticus*

### Bacteria from water samples

The prevalence of bacteria in water samples from Lake Hawassa was 50%, Ziway 33.3% and Langanoo 16.7% with significant difference (*p* = 0.046). *A****.**** sobria, C****.**** koseri, S****.**** paratyphi, S****.**** flexneri* and *V****.**** parahemolyticus* were detected in Lake Hawassa. The only bacteria detected in Lake Langano and exclusive to it were *C****.**** freundii* and *K****.**** pneumonia.* Similarly, from 4 species in Lake Ziway, 3 (*E****.**** coli*, *P****.**** aeruginosa*, *S****.**** dysenteriae*) were exclusive to it (Supplementary Table 4). Not only in terms of prevalence but bacterial species diversity as well, Lake Hawassa was the most diverse and Langanoo the least.

## Discussion

The mean values of all the measured physicochemical parameters in the three lakes were within the standard limit for fish health [24–29]. In light of this, the lakes could be considered fitting for fish survival. Water quality factors such as DO, temperature, ammonia, phosphate, pH, alkalinity, hardness and clarity affect fish health in multiple ways. Each water quality factor interacts with and influences other parameters, sometimes in complex ways and what may be fatally toxic in one situation can be harmless in another. Nevertheless, we did not test many other parameters, which are important water quality factors for logistics reasons. The effect of sampling site, season and hour on the parameters is usually considerable. For instance, DO values significantly vary when the sampling site is surface water and along shorelines compared to deep water, or at the center. Our sampling was on surface water.

The oxygen requirements of fish also depend on a number of other factors including temperature, pH, and CO_2_ level of the water, as aforementioned, and the metabolic rate of the fish. Therefore, changes in these physicochemical parameters in the aquatic environment are primary causes of fish stress [27] although the health impact of such stress may depend not only on the severity of the stress, but on its duration and the fish's overall physiological status.

The mean DO values of 6.27 mg/l (Lake Hawassa), 5.10 mg/l (Lake Langano) and 3.93 mg/l (Lake Ziway) indicate relatively better aeration at lakes Hawassa and Langanoo than Ziway at least during the study period and sampling time. This might be attributable to variations in temperature and flow rate in the Lakes during the sampling season as lower temperature and good flow rate are associated with higher ODs [24, 30]. Most DO in ponds is produced during photosynthesis by aquatic plants and algae. For this reason, DO increases during daylight hours, declines during the night, and is lowest just before daybreak. DO concentrations below 5 mg/l may be harmful to fish and piping (gulping air at the surface) may be observed when DO falls below 2 mg/l. Low levels of DO are most frequently associated with hot, cloudy weather, algae die-offs, or heavy thunderstorms [30]. Overall, poor water quality is a key factor for low fish yields. In a pond study, whereas increase in temperature and OD was correlated with tilapia growth rate, increase in conductivity and pH showed the opposite [31]. Different fish species have different requirements for water DO concentration [27], and water temperature may influence fish feeding, growth and overall behavior.

Similarly, the data showed insignificant pollution by nitrogenous wastes at the sampling time although ammonia is a pollutant frequently found in aquatic ecosystems. In fish, ammonia can cause physical damage, alter its behavior such as lower swimming activity and feeding behavior, and oxidative stress response, and even cause death. Exposure to ammonia also increases fish physiological stress and recent evidence suggested that once exposed, fish suffer from reduced antioxidant defenses and thus increased oxidative tissue damage even if water quality was improved [32].

The only exception was phosphate level in Lake Langano which was relatively high (2.34–4.48 mg/l). The highest concentration of phosphate (4.48 mg/l) recorded for Lake Langano *O’etu* site (*S2*) was higher than the standard limit although the mean value was normal. The mean value of phosphate for Langanoo was substantially higher than that of Hawassa, and that of Ziway was much lower. In the early 1990s, it was reported that Lakes Ziway and Hawassa (then Hawassa) were phosphorus-limited, whereas Langanoo has surplus phosphorus [33]. It appeared that Lake Langano remained phosphate surplus. In the 1960s, however, increases in phosphorus in the lower Lakes raised considerable public concern [34]. The augment in phosphorus could be due to increased surface runoff from phosphate containing fertilizers and certain industrial wastes. Phosphate is an essential plant nutrient. However, high phosphate level can lead to eutrophication boosting plant/algae growth. These plants/algae eventually decay and cause DO depletion in the water threatening certain species of fish favoring phosphate-pollution-tolerant organisms.

Although it appeared that the physicochemical parameters of the Lakes were normal, the proportion (20%) of fish with visible health problems is not a good sign. Alternatively, it may be plausibly argued that encountering relatively lesser number of clinically diseased fish with visible pathological changes might be attributed to the good water quality of the Lakes during the study period notwithstanding the seasonal fluctuations in water quality [35, 36]. At least during the study season, majority of the fish caught were less stressed. The effect of stress on freshwater fish may be a factor of the severity of the stress, its duration and the overall physiological state of the fish [37].

The proportion of bacterial isolates from the intestine of live-caught fish (17.6%) was significantly higher than that of the liver (12.4%) or kidney (9.1%). But, there were slight variations with respect to the individual bacteria species. For instance, the number of isolates from live-caught fish intestine was equal to that of the liver concerning *E. coli* which was the most prevalent bacterium isolated. The second most prevalent in this study, *E. tarda*, was similarly isolated from the intestine and liver in equal proportions. *E. tarda* is a cause of rare but fatal food-/waterborne infection in man [38]. Contrarily, *E. aerogenes* was most frequently isolated from the kidney. A study on marine fish found significantly higher potential pathogenic bacteria in kidneys than in liver samples and a variation was found between the fish species [39]. The authors reported that significant differences were observed between fish species, organs and sites, indicating the importance of the environmental conditions on the fish microbiome. Although we did not investigate bacteria in different portions of the fish gut, it appears that microenvironment dynamics along the gut of various fish species may influence the composition and abundance of the bacterial flora [40].

In African catfish, higher bacterial load was recovered from the intestine than other organs like skin and gills [41]. There is evidence that dense microbial populations occur within the intestinal contents, with numbers of bacteria much higher than those in the surrounding water, like the current finding, indicating that the intestines provide favorable ecological niches for these organisms [42]. However, the method of intestinal bacteria sampling varied in different surveys. Some used anal swabs, others only the intestinal contents, while still in others intestinal tract and contents were homogenized and used for culturing [43]. Austin and Al-Zahrani (1988) [44] distinguished between the flora of the gut contents and that intimately associated with the wall of the gastrointestinal tract, and noted that scanning electron microscopy showed only sparse microbial colonization of the wall. Although the genera present in the gut generally seem to be those from the environment or diet which can survive and multiply in the intestinal tract, there is evidence for a distinct intestinal microflora in some species [43]. This same author reviewed the progressive decline in the numbers of aerobic heterotrophic bacteria along the digestive tract. Anaerobes were detected only in the upper intestine and in the intestinal contents.

However, other investigators [45] found that numbers of bacteria in freshwater salmonids increased between the stomach and the posterior portion of the intestine. The authors suggested that these numbers must represent active multiplication in the tract, as they could not be accounted for by ingestion. The numbers detected in this survey were probably an artificially low estimate since the methods used did not allow for the isolation and growth of strict anaerobes, species sensitive to oxygen, nutritionally fastidious species, or those requiring low growth temperatures (< 20 °C). In addition, the counts obtained were based on the total tissue weight in each sample, while the bacteria actually populate only the epithelial surface of the tract, and the rest of the tissue sterile. There was no significant difference in the bacterial flora of fish of different species, sex, breeding status, weight, or geographical source. But, microenvironment characteristics at various locations through the gastrointestinal tract of fish influence the composition and abundance of gut bacteria [46] and bacterial counts significantly differed between species, sources and feeding habits of examined fishes [47].

Fish internal organs such as the spleen, liver, and kidneys are expected to be sterile [48]. Nonetheless, evidence accumulates for bacteria from internal organs of apparently healthy fish [49] in agreement with the current study. Possible fish immune compromise may explain such observations. Immune defect could happen due to stress. Fish stress could be associated with poor water quality, temperature changes, nutritional deficiencies, overcrowding, trauma, parasitism, primary viral infections [50–52], among others. For instance, fish immunity was found significantly affected by lower temperatures [53] making them susceptible to obligate or facultative pathogenic bacteria such as *A. hydrophila*.

The bacterial flora of the gut of two marine fish has been investigated in an attempt to clarify the relationship between these bacteria and the bacterial flora of their diets, and to determine the effect of the degree of specialization of the digestive tracts on their floras [54]. Bacterial flora of fish with relatively undeveloped digestive tracts reflected that of the fishes' food, whereas fish with more specialized tracts have a distinctive gut microflora. In the red sea bream, the composition of the bacterial floras of the stomach and intestine changed with time after feeding. Half an hour after ingestion, most of the bacteria isolated from the stomachs resembled those of the fish meat diet, but after 6 h, vibrios resistant to bile and low pH predominated. Further work comparing representative strains of these indigenous vibrios from the red sea bream with other isolates from the stomach and intestine showed that the vibrios were able to survive in the presence of gastric juice at pH 4, and were able to grow, although at a reduced rate, at pH 5, while most of the other isolates were inhibited by these conditions [55].

In this study, the water samples were positive for *A. sobria, Citrobacter* spp., *E*. *tarda, E. coli, K. pneumoniae, P. aeruginosa, S. typhi, S. dysenteriae* and *S. flexneri.* Detection of these and other related bacteria both in marine and freshwater habitats and fish has been widely recorded [56–59]. *Aeromonas* is associated with a range of human opportunistic infections including enteritis and septicemia [60–62] and is one of the most common pathogens in tropical fish [63]. *E. coli* was the most frequently detected bacterium in processed and live-caught fish samples in this study. Total and fecal coliforms such as *E. coli* are indicators of fecal contamination of aquatic environments and food. Detection of *E. coli* in processed fish samples could be due to unhygienic handling during processing.

Moreover, the fact that most of *E. coli* isolated during this study was from fish intestine reflecst warm-blooded animal pollution level of the water. *E. coli* can have a long-term survival and can multiply depending on fish and water temperatures [64–67]. It is known that fish possess distinct intestinal microbiota, but nutritional status or feeding habits, trophic level, species (attribute to complexity of the fish digestive system) and the environmental conditions (salinity of the habitats and the bacterial load in the water) are the most influential factors which change the intestinal microbiota composition and abundance [57]. This may explain the observed differences in the distribution of bacteria from fish samples in the three Lakes. While bacteria in water can influence the microbial flora associated with fish, the reverse is also worth consideration.

*Shigella* spp. and *Salmonella* spp. are pathogenic bacteria found in animal, human or environmental reservoir. Although contamination of fish products with these bacteria is commonly from the environment, their incidence in ready-to-eat fish product due to unhygienic handling cannot be ruled out [68]. *Citrobacter*, *Enterobacter* and *Klebsiella* are indigenous to general environment and frequently present in fish but most of these bacteria are considered non-pathogenic environmental strains. The bacterial species isolated from processed fish (*E. coli*, *K. pneumonia* and *S. paraTyphi*) were also recovered from the water samples.

The variation in the number of isolates and bacterial species between sampling sites of the study Lakes might be attributed to the relative distance and degree of exposure to the nearby point source pollution around the study area. Disruption of the environmental microbiome after an earthquake followed by seasonal variation in the water quality was noted although restoration of these microbial communities as a function of time and sanitation practices occurred in Nepal [69].

All positive live fish samples were positive for at least one isolate of all the 15 bacteria species recovered. This shows the higher bacteria species diversity in fish compared to the aquatic environment wherein only 4 species, *E. aerogenes, E. cloacae, P. shigelloides* and *S. typhi*, were characterized although the water bacteria prevalence was higher. The finding suggests that the bacteria detected in fish internal tissue might constitute the natural fish microbiota and/or the fish bacteria source might be their diet. Alternatively, the fish might have been exposed to seasonal or occasional biological pollutants which have been diluted or neutralized from the environment. Such dynamics in the aquatic ecosystem may explain particularly the absence of human urinary and respiratory tracts pathogens *E. aerogenes* and *E. cloacae* [70] in water samples and their detection in fish. Another surprising finding is the absence of *P. shigelloides* in the water samples. The common environmental reservoirs for this organism include freshwater ecosystems and estuaries and inhabitants of these aquatic environs, and a series of foodborne enteritis outbreaks have been solely or partially attributable to *P. shigelloides* [71]. Another species that is persistently detected in freshwater environments and stands among major causes of food-/waterborne human illnesses [72], but which could not be detected in the water samples of the current Lakes was *S. typhi.*

Detection of the common fecal coliforms (*E. coli, K. pneumonia, E. aerogenes*) and *Salmonella* spp. in all of the sample types especially in processed fish, signals the danger of passage of these pathogens and their toxins to man via infected and contaminated fish products. *Salmonella* spp. and fecal coliforms were detected in 42% of water samples and 64% of processed fish samples in this study. *Shigella* spp. and *Salmonella* spp. are pathogenic bacteria found in animal or human reservoir and contamination of fish products by these bacteria is almost always due to poor hygiene.

Except *A. sobria*, all the others species were detected from tilapia in different frequencies. Similarly, all bacteria species were also detected from catfish with the exception of *P. shigelloides.* However, *C. freundii, C, koseri* and *V. parahemolyticus* were not isolated from carps. Even though it seemed that some bacteria species tended to be specifically associated with a particular fish species, the association was not statistically significant. Different studies reported the occurrence and antimicrobial resistance of *A. sobria, E. tarda, P. shigelloides, P. aeruginosa, Citrobacter* spp. and *Klebsiella* spp. from tilapia and catfish [73–76].

During the study period, differences were observed in the bacterial prevalence and frequency across the sampling sites of each Lake. This may be attributed to the relative distance and degree of exposure to a nearby pollution source around.

Although the report on fish bacteria and their occurrence in humans is limited in Ethiopia, there were some efforts. Among the bacteria found in this study, *E. coli, Klebsiella* spp., *Enterobacter* spp., *Citrobacter* spp., and *Aeromonas* spp. which are enterotoxin-producing were detected in stools of Ethiopian children with diarrhoeal disease in the late 1970s [77]. *E. tarda* was isolated from the liver of a tilapia from Lake Ziway for the first time for the Lake [15]. The other bacteria detected in this same study were *E. coli*, *Kebsiella oxyloca*, *Citrobacter* spp. and *Yersinia enterocolitica.* Another investigator [78] isolated *Aeromonas* spp. including *A. sobria*, *E. tarda*, *Vibrio* spp., *E. aerogenes P. shigelloides, E. coli*, *K. pneumoniae*, *Shigella* spp., *Citrobacter* spp. from fish of Lake Tana. The author also reported that all the bacterial species, which were isolated from the water samples, were also recovered from fish in the Lake. Moreover, an outbreak of *A. hydrophila* associated with a certain parasite in pond of African catfish fingerlings at Sebeta, central Ethiopia, was reported [79]. *Vibrio* spp., *Salmonella, Shigella* and *E. coli* were detected from surface water and sediment samples of Lake Ziway and drinking water system of Batu (former Ziway) town, Ethiopia [80].

From the total 410 fish samples examined, six were found contaminated with Shiga toxin-producing *E. coli* strain in Ethiopia [81]. The isolates were resistant to ampicillin and streptomycin disks. However, ciprofloxacin, gentamicin and nalidixic acid were found effective in inhibiting the growth of all of the isolates. *Vibrio*, *Escherichia*, *Aeromonas*, *Pseudomonas, Salmonella* and *Streptococcus* were detected from Nile tilapia in Hawassa with the bacterial population significantly higher in the intestine than in the liver [82]. A short review on bacterial pathogens of fish presented pathogenic and zoonotic bacteria such as *Edwardsiella*, *Salmonella, Escherichia, Staphylococcus, Vibrio* and *Aeromonas* recovered from fish from various parts of Ethiopia [83]. A more recent molecular study that analyzed the diversity of microbiota in different sections of tilapia gut found more diversity in Lake Chamo than Lake Hawassa [40].

In some countries like Poland [84] and Malaysia [85], the emergence of hitherto unreported pathogenic fish bacteria is becoming evident. Thus, fish bacteria detection methods in Ethiopia must take into account less known and unreported ones as well. Moreover, exploring possible reciprocal transmission of potential pathogenic bacteria from wild fish to aquaculture, and domestic animals or humans is essential. This will contribute towards microbial-source-detection investigations.

This work will serve as an initial step to establish a baseline dataset of microbial communities associated with wild freshwater fish in Ethiopia. But, it has certain notable limitations. It neither quantified the detected bacteria, nor molecularly identified them, and no antibiotic susceptibility test was done. The results would have been more robust if samples from fish skin and gills which are gateway routes of transient or resident microbiota and/or potential pathogenic bacteria have been included. Moreover, the study did not assess seasonal patterns of both water quality and fish microbiota.

## Conclusion

Despite the fact that the physicochemical parameters of the water samples were within normal range at which most freshwater fish are non-stressed; the 20% prevalence of clinically sick fish is a source of concern. Moreover, the bacteria identified from water, live fish and processed fish samples are potential pathogens of fish and man, spoilage agents and indicators of environmental contamination. In processed fish particularly, hygiene indicator bacteria occurred at higher level suggesting that fresh processed fish available in fish markets of Batu town likely act as a reservoir of pathogenic bacteria. Moreover, the identified bacteria species are zoonotic and associated with food-borne illnesses. This has implications for the fish market and consumer health. Therefore, consistent adherence to simple hygienic steps is advisable. Further, establishing a practice of regular inspection of fish products and source aquatic habitats, including fish processing channels for pathogenic bacteria and environmental sanitation is indispensable.

## Supplementary Information

**Additional file 1:** **Supplementary Table 1.** Morphometric and other characteristics of Lakes Ziway, Langanoo and Hawassa. **Supplementary Table 2.** Mean ± SD values of physicochemical parameters of four different sampling sites (S1-S4) of Lakes Hawassa, Langanoo and Ziway where from water samples were drawn DO: dissolved oxygen, EC: electrical conductivity. **Supplementary Table 3.** Number (percentage) of bacteria isolated from clinically sick live-catch fish from Lakes Hawassa, Langanoo and Ziway (*N* = 36). **Supplementary Table 4.** Distribution of bacteria from water samples of Lakes Hawassa, Langanoo and Ziway (*N* = 12).

**Additional file 2:** **Supplementary Figure 1.** Sample microscopic morphology of the isolated isolates. **Supplementary Figure 2.** Samle colony morphology of bacterial  isolates on XLD agar plate.

## Data Availability

All data and materials are within this published paper.
