# Supplementary material for: Isolation and identification of major bacteria from three Ethiopian rift valley lakes live and processed fish, and water samples: implications in sanitary system of fish products
Source: BMC Vet Res. 2022 Dec 14;18:439. doi: 10.1186/s12917-022-03508-w (PMC9749233; doi:10.1186/s12917-022-03508-w)
Supplement: Supplementary file 1 — Additional file 1: Supplementary Table 1. Morphometric and other characteristics of Lakes Ziway, Langanoo and Hawassa. Supplementary Table 2. Mean ± SD values of physicochemical parameters of four different sampling sites (S1-S4) of Lakes Hawassa, Langanoo and Ziway where from water samples were drawn DO: dissolved oxygen, EC: electrical conductivity. Supplementary Table 3. Number (percentage) of bacteria isolated from clinically sick live-catch fish from Lakes Hawassa, Langanoo and Ziway (N = 36). Supplementary Table 4. Distribution of bacteria from water samples of Lakes Hawassa, Langanoo and Ziway (N = 12). [file 12917_2022_3508_MOESM1_ESM.docx]

Supplementary Table 1: Morphometric and other characteristics of Lakes Ziway, Langanoo and Hawassa

| Description | Ziway | Langanoo | Hawassa |
| --- | --- | --- | --- |
| Altitude (m) | 1636 | 1852 | 1680 |
| Latitude | 7.93333 | 7.5932 | 7.0313 |
| Longitude | 38.71667 | 38.7578 | 38.4219 |
| Shoreline (km) | 102 | 7.5 | 52 |
| Length (km) | 31 | 18 | 16 |
| Width (km) | 20 | 16 | 9 |
| Surface area (km^2^) | 442 | 241 | 129 |
| Catchment area (km^2^) | 7025 | 1600 | 1300 |
| Mean depth (m) | 2.3 | 0.7 | 10.7 |
| Maximum depth (m) | 8.95 | 47.9 | 21.6 |
| Volume (km^3^) | 1.6 | 5.3 | 1.34 |
| Salinity (g/L) | 0.349 | 1.88 | 1.008 |

Supplementary Table 2: Mean ± SD values of physicochemical parameters of four different sampling sites (S1-S4) of Lakes Hawassa, Langanoo and Ziway where from water samples were drawn

| Description | | | | | | | | | | |
| --- | --- | --- | --- | --- | --- | --- | --- | --- | --- | --- |
| Hawassa Samples | pH | | Temp., °C | DO, mg/l | EC, µS/cm | Nitrate, mg/l | | Phosphate, mg/l | | |
| S1 | 8.86±0.04 | | 24.6±0.10 | 5.66±0.05 | 1961±1.90 | 4.33±0.40 | | 1.31±0.10 | | |
| S2 | 9.12±0.05 | | 24.1±0.10 | 6.84±0.20 | 2156±1.40 | 4.37±0.50 | | 1.44±0.04 | | |
| S3 | 5.74±0.04 | | 25.8±0.80 | 6.78±0.40 | 741±3.10 | 3.12±0.20 | | 1.96±0.03 | | |
| S4 | 7.92±0.10 | | 28.7±0.10 | 5.82±0.90 | 1836±2.60 | 3.72±0.40 | | 0.42±0.06 | | |
| Average | 7.91±0.10 | | 25.8±0.80 | 6.27±0.90 | 1673.5±487 | 3.88±0.60 | | 1.28±0.90 | | |
| Langanoo | | | | | | | | | | |
| S1 | | 9.14±0.04 | 24±0.51 | 4.99±0.18 | 1522±3.20 | | 2.46±0.27 | | 2.92±0.41 | |
| S2 | | 9.40±0.05 | 26±0.48 | 5.47±0.21 | 1717±4.90 | | 2.83±0.31 | | 4.48±0.27 | |
| S3 | | 6.02±0.04 | 19±0.39 | 4.79±0.35 | 902±1.80 | | 2.44±0.28 | | 2.46±0.29 | |
| S4 | | 8.20±0.10 | 22±0.54 | 5.16±0.59 | 1647±7.40 | | 2.31±0.26 | | 2.34±0.31 | |
| Average | | 8.19±0.09 | 22.75±0.43 | 5.10±0.54 | 1447±5.70 | | 2.51±0.29 | | 3.65±0.44 | |
| Ziway | | | | | | | | | | |
| S1 | | 8.61±0.12 | 23±0.49 | 4.32±0.31 | 281±11 | | 0.17±0.12 | | | 0.63±0.86 |
| S2 | | 8.84±0.58 | 22±0.56 | 4.10±0.22 | 362±92 | | 0.48±0.04 | | | 0.76±0.06 |
| S3 | | 8.60±0.09 | 22±0.28 | 2.8±0.30 | 236±43 | | 0.96±0.24 | | | 0.97±0.08 |
| S4 | | 8.67±0.10 | 23±0.11 | 4.5±0.28 | 279±56 | | 0.25±0.11 | | | 0.21±0.04 |
| Average | | 8.68±0.11 | 22.50±0.36 | 3.93±0.18 | 289.50±14 | | 0.46±0.14 | | | 0.64±0.81 |

DO: dissolved oxygen, EC: electrical conductivity

Supplementary Table 3: Number (percentage) of bacteria isolated from clinically sick live-catch fish from Lakes Hawassa, Langanoo and Ziway (N = 36)

|  | LH | | | LL | | | LZ | | |  |  |
| --- | --- | --- | --- | --- | --- | --- | --- | --- | --- | --- | --- |
| Bacteria sp | carp | catfish | Tilapia | carp | catfish | tilapia | carp | catfish | tilapia | Total | P-value |
| *A. sobria* | 2(5.6) | - | 1(2.8) | - | - | - | - | - | - | 3(8.3) | 0.154 |
| *E. tarda* | 2(5.6) | - | 5(13.9) | - | - | - | - | - | - | 7(19.4) | 0.042* |
| *Ps* | - | - | - | - | - | - | 2(5.6) | - | 1(2.8) | 3(8.3) | 0.106 |
| *Pa* | - | - | - | - | - | - | 3(8.3) | - | 1(2.8) | 4(11.1) | 0.102 |
| *Sp* | 1(2.8) | - | 1(2.8) | - | - | - | 1(2.8) | - | 1(2.8) | 4(11.1) | 0.632 |
| *S. Typhi* | 1(2.8) | - | 1(2.8) | - | - | - | 1(2.8) | - | 1(2.8) | 4(11.1) | 0.632 |
| *Sd* | - | - | 1(2.8) | - | - | - | 1(2.8) | - | 1(2.8) | 3(8.3) | 0.578 |
| *S*. *flexneri* | 2(5.6) | - | 1(2.8) | - | - | - | 1(2.8) | - | 1(2.8) | 5(13.9) | 0.768 |
| *Vp* | - | - | 1(2.8) | - | - | - | - | - | 2(5.6) | 3(8.3) | 0.078 |
| Total | 8(22.2) | - | 11(30.6) | - | - | - | 9(25) | - | 8(22.2) | 36(100) | 0.062 |

-: Not detected, *Sd: S. dysenteriae, Sp: S. paraTyphi, Pa: P. aeruginosa, Ps: P. shigelloides, Vp: V. parahemolyticus,* LH: Lake Hawassa, LL: Lake Langanoo, LZ: Lake Ziway

Supplementary Table 4: Distribution of bacteria from water samples of Lakes Hawassa, Langanoo and Ziway (N = 12)

| Bacteria species | Lakes | | |  |  |
| --- | --- | --- | --- | --- | --- |
|  | Hawassa, n(%) | Langanoo, n(%) | Ziway, n(%) | Total, no(%) | P-value |
| *A****.*** *sobria* | 1(16.7) | - | - | 1(8.3) | 0.064 |
| *C****.*** *freundii* | - | 1(50) | - | 1(8.3) | 0.087 |
| *C****.*** *koseri* | 1(16.7) | - | - | 1(8.3) | 0.076 |
| *E****.*** *tarda* | 1(16.7) | - | 1(25) | 2(17) | 0.108 |
| *E****.*** *coli* | - | - | 1(25) | 1(8.3) | 0.066 |
| *K****.*** *pneumoniae* | - | 1(50) | - | 1(8.3) | 0.102 |
| *P****.*** *aeruginosa* | - | - | 1(25) | 1(8.3) | 0.072 |
| *S****.*** *paraTyphi* | 1(16.7) | - | - | 1(8.3) | 0.098 |
| *S****.*** *dysenteriae* | - | - | 1(25) | 1(8.3) | 0.072 |
| *S****.*** *flexneri* | 1(16.7) | - | - | 1(8.3) | 0.064 |
| *V****.*** *parahemolyticus* | 1(16.7) | - | - | 1(5.3) | 0.088 |
| Total | 6(50) | 2(16.7) | 4(33.3) | 12(100) | 0.046* |
