# Supplementary material for: Isolation and identification of major bacteria from three Ethiopian rift valley lakes live and processed fish, and water samples: implications in sanitary system of fish products
Source: BMC Vet Res. 2022 Dec 14;18:439. doi: 10.1186/s12917-022-03508-w (PMC9749233; doi:10.1186/s12917-022-03508-w)
Supplement: Supplementary file 2 — Additional file 2: Supplementary Figure 1. Sample microscopic morphology of the isolated isolates. Supplementary Figure 2. Samle colony morphology of bacterial isolates on XLD agar plate. [file 12917_2022_3508_MOESM2_ESM.docx]

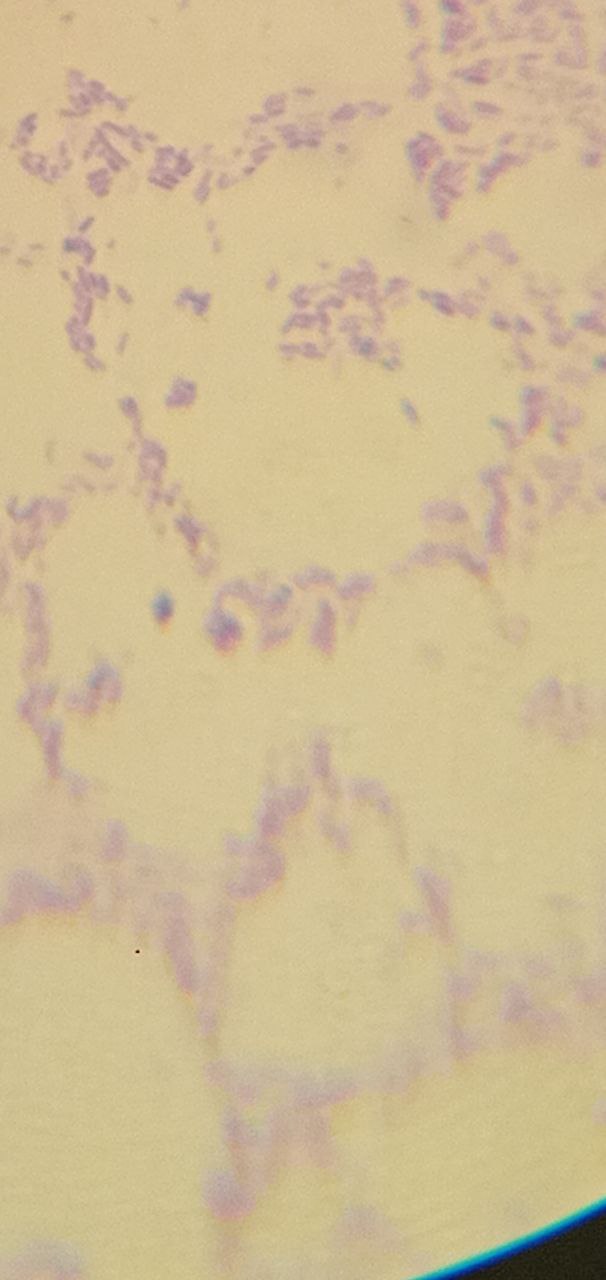

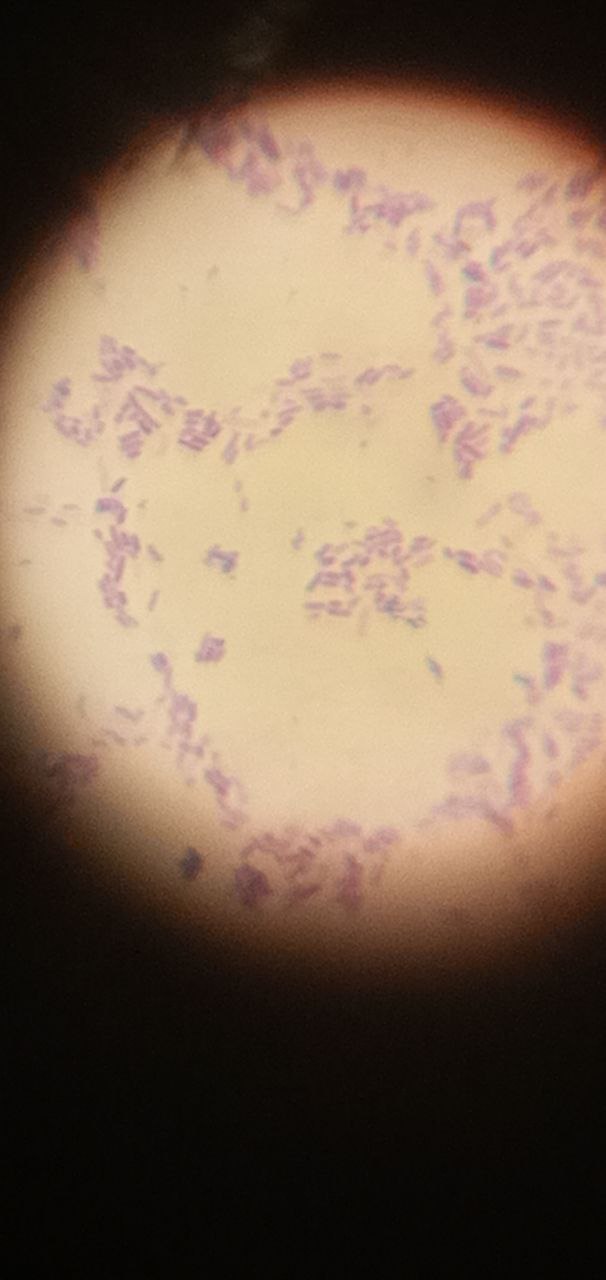

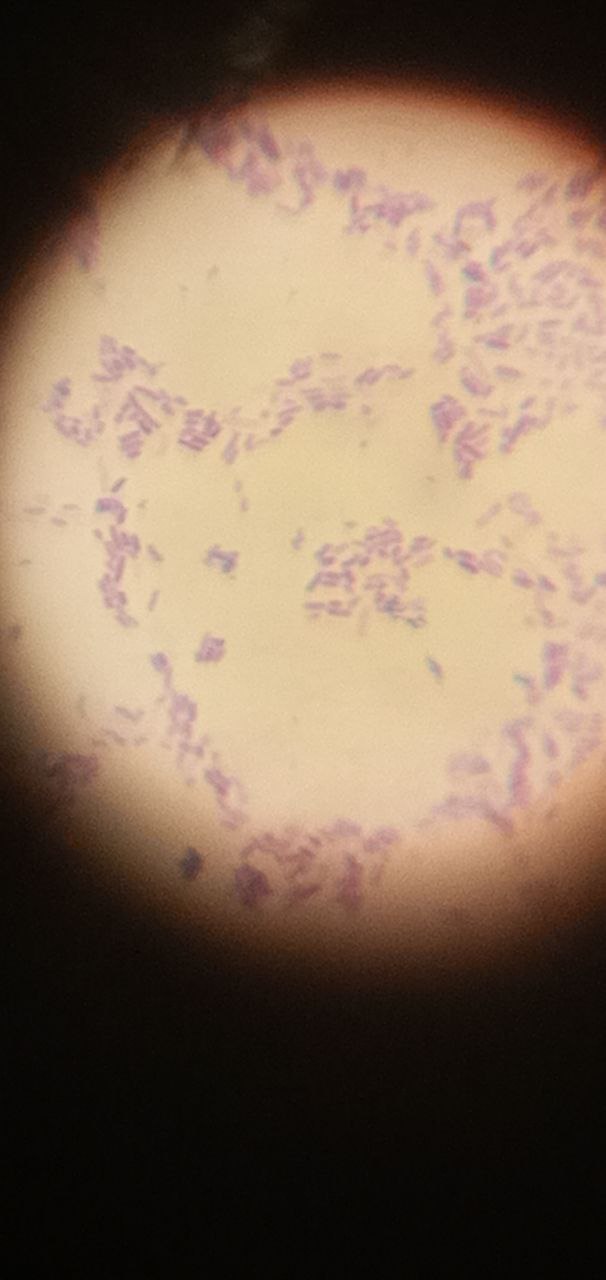


Supplementary Figure 1: Sample microscopic morphology of the isolated isolates


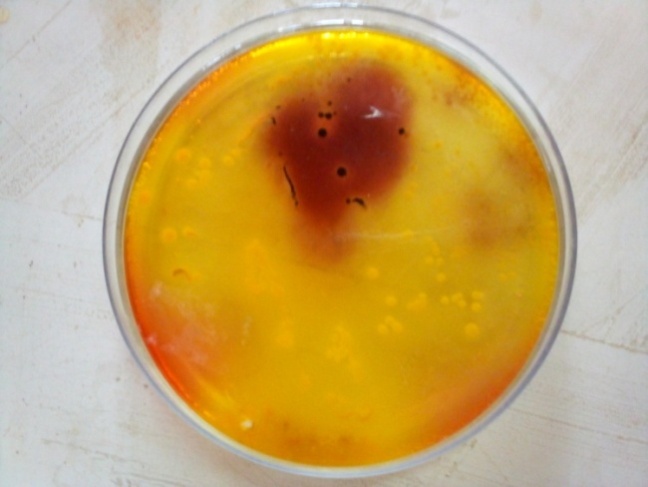

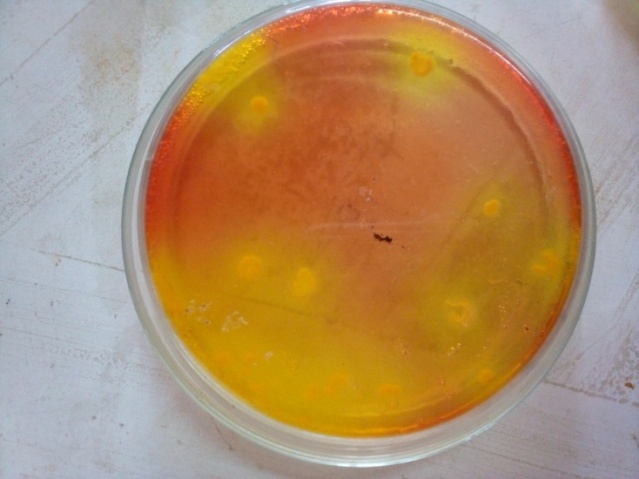

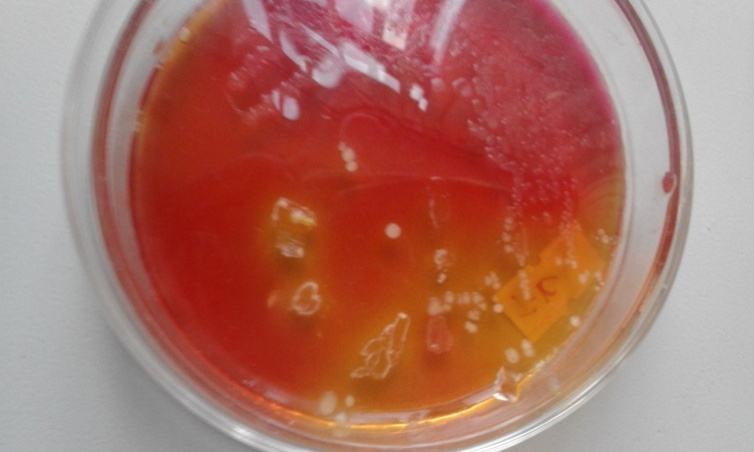


Supplementary Figure 2: Samle colony morphology of bacterial isolates on XLD agar plate
